# Escaping negative moods and concentration problems play bridge roles in the symptom network of problematic smartphone use and depression

**DOI:** 10.3389/fpubh.2022.981136

**Published:** 2023-01-17

**Authors:** Xinyi Wei, Fei An, Chang Liu, Kuiliang Li, Lin Wu, Lei Ren, Xufeng Liu

**Affiliations:** ^1^Department of Psychology, Renmin University of China, Beijing, China; ^2^Department of Military Medical Psychology, Air Force Medical University, Xian, China; ^3^BrainPark, School of Psychological Sciences, Turner Institute for Brain and Mental Health, Monash University, Clayton, VI, Australia; ^4^Department of Developmental Psychology of Armyman, Department of Medical Psychology, Army Medical University, Chongqing, China

**Keywords:** smartphone addiction, depression, network analysis, central symptoms, bridge symptoms

## Abstract

**Background:**

Problematic smartphone use (PSU) has become an issue of great concern in the age of smartphones. PSU is associated with emotional problems, one of which is depression, as shown by empirical studies. However, previous studies have been limited in that they have focused solely on the total score for symptoms of PSU and depression while ignoring the symptomatic heterogeneity of these two concepts.

**Method:**

This study filled this gap by exploring links between symptoms of PSU and depression among 1,849 university students (59.17% female participants, 17–23 years old). Network analysis was utilized to reveal symptom connections, central symptoms, and bridge symptoms between PSU and depression.

**Results:**

(1) A total of 17 of 81 symptom connections (about 21%) between PSU and depression existed in the symptom network. For example, “self-control failure” for PSU was positively correlated with “concentration problems” for depression; (2) “recklessly continuing” for PSU and “fatigue” for depression were central symptoms within the PSU symptom network and depression symptom network, respectively; (3) “escaping negative moods” for PSU and “concentration problems” for depression were bridge symptoms. The former was maximumly connected with the depression symptoms and the latter was maximumly connected with the PSU symptoms; and (4) gender had very minimal influence on the network characteristics.

**Conclusion:**

The results are in keeping with the central idea of the compensatory internet use theory that excessive smartphone use may be a coping strategy for depressed emotions derived from escaping motivation. Moreover, concentration problems may be a mediator explaining how negative emotions (e.g., depression) cause PSU, which is undefined in current internet use theories. Finally, symptom connections, central symptoms, and bridge symptoms could be potential targets for the prevention and intervention of PSU and depression in young adults.

## 1. Introduction

Depression is associated with serious consequences that hinder emerging adult development, such as smoking and alcohol problems, poor sleep quality, and suicidality (1–3). Depression is especially prevalent among young adults in university, with a prevalence of 25% shown in a recent meta-analysis (4). Over the last decade, the prevalence of depression has increased among young adults, accompanied by an increase in problematic smartphone use (PSU) (5, 6). Compared with other internet devices (such as computers), smartphones have the characteristics of portability and availability (7). PSU may bring about some negative effects, such as anxiety, negative self-disclosure, impaired academic performance/family life/interpersonal relationships, and stress (8). Thus, people may be prone to no control of their use and thus experience negative moods; furthermore, people with a high tendency toward negative moods may tend to use smartphones to escape such moods. In previous systematic reviews and meta-analyses, the effect size of the link between PSU and depression was small to medium (9, 10).

In previous studies, many researchers have used total scores for PSU and depression, rather than dimension/item scores. The total PSU score from different instruments may not be an appropriate indicator for assessing different constructs of PSU. This is because the total PSU scores from current different instruments may only evaluate the addiction structure of PSU. A review of previous PSU measurement tools found that although some existing scales include the non-addiction structure of PSU, the measurement objectives and most topics of the existing scales are aimed at measuring the addiction construct of PSU (11). Some addiction psychologists emphasize that PSU has two main different constructs: the addiction construct and the non-addiction construct (characterized by escapism behaviors through smartphone use) (12, 13). Furthermore, the addiction construct consists of different symptom constructs (e.g., self-control failure symptom, withdrawal symptom, and tolerance symptom) (12, 13). Similarly, depression includes different symptoms such as depressed moods, sleep problems, and fatigue (1, 2, 6). This indicates that it is necessary to analyze different symptoms of PSU and depression. To the best of our knowledge, little is known regarding symptom connections, central symptoms, and bridge symptoms between PSU and depression. Therefore, an interesting but not yet resolved issue could be exploring the link from a symptom-level perspective.

One approach to solving this issue is network analysis (NA), which could reveal the link between symptoms/items (14). Applying NA to this research can be appropriate and has some scientific importance. First, in the NA perspective, interrelationships between symptoms are permitted; thus, potential causal pathways between symptoms may emerge. This approach is like a type of psychotherapy that analyzes the causes and consequences of psychology and behaviors (14). For example, the hopelessness-related symptoms of depression logically may enhance the suicide symptom of depression. However, in the traditionally observed variable or latent variable model, the link between symptoms is set to be irrelevant (15). Second, the current understanding of the mental disorder has been restricted to the pathway/correlation between variables, instead of the severity of each symptom (14). NA could provide some novel and informative indexes of each symptom from a psychological network perspective. “Node expected influence” or “node centrality” can reveal the extent to which a symptom is associated with all other symptoms and may thus reveal the centrality/importance of the symptom in the psychological network (15). Moreover, the “bridge node expected influence” or “bridge centrality” index can reveal a symptom in one community that maximally connects to other symptoms in another community (15); therefore, it may be useful to explain why the two disorders are co-occurrent (16). However, given the lack of network studies focusing on this link, applying these new indexes to understand the link is urgently needed.

Although some studies have explored the link between internet use and emotional problems (17, 18), to our knowledge, only two prior network analysis studies have partly been involved in the link between PSU and depression. Andrade et al. (19) examined the link between five facets of smartphone use (i.e., “harm of smartphone uses on everyday life”; “rate of mobile time use” and “time spent on the smartphone”; “smartphone checks daily”; “number of messages sent daily”; and “number of messages received daily”) and global depressive severity; all facets were unrelated to depressive severity. However, their measure includes only one PSU symptom (i.e., “harm-everyday-life”); heterogeneity of depression is neglected because a global level (i.e., total score) of depression is used, making the claim of no significant link uncertain. Mancinelli et al. (20) explored the relationship between the global level of PSU, the global level of internalizing problems, and an item of self-harming using network analysis. PSU was not associated with internalizing problems and self-harming; however, internalizing problems and self-harming are not the same as depression. Moreover, these variables were also not distinguished as different symptoms. In the domain of internet addiction, a recent network analysis uncovered a link between symptoms of internet addiction and depression; it suggested that preoccupation, neglect of chores, and tolerance of internet addiction and guiltiness were central depression symptoms (21). However, their findings cannot be generalized to PSU because internet addiction and PSU are two distinct concepts; more than 50% of the symptoms of internet addiction and all symptoms of PSU were not significantly associated (22). Previous studies also found that PSU had a stronger link with depression than internet addiction (23), but there has been no network analysis analyzing this link at the symptom level. Therefore, we aimed to deepen our understanding of this link using item-/symptom-level network analysis.

The aims of this study are as follows: (a) to uncover the edges/pathways between PSU symptoms and depression symptoms; (b) to identify central symptoms of PSU and depression that maximumly link other symptoms in the network of these two concepts; (c) to identify bridge symptoms of PSU and depression that maximumly connect with a community of depression symptoms and a community of PSU symptoms, respectively; and (d) to explore whether gender has an influence beyond network characteristics, given the female susceptibility to depression and PSU (24, 25).

## 2. Method

### 2.1. Ethics statement

The data collection procedure followed the Declaration of Helsinki and was approved by the Ethics Committee of the First Affiliated Hospital of the Fourth Military Medical University.

### 2.2. Participants

Participants were undergraduate students who were conveniently sampled from five universities in western China. Although they were not patients with PSU and depression in hospital, this did not affect our study in terms of exploring the relationship between PSU and depression. The significant relationship between PSU and depression is a common phenomenon among the non-clinical population (26, 27). Well-trained investigators informed participants regarding anonymity, confidentiality, the voluntary nature of participation, and their right to withdraw at any time. All participants were provided informed consent. A total of 176 participants were excluded due to failing the two honesty check items. The inclusion criteria are as follows: (1) having at least one smartphone device for free use and at least 1 year of smartphone user experience; and (2) consent to participate in the study. The exclusion criteria are as follows: failing the two honesty check items (e.g., participants did not choose the second option when they responded to “to make sure your answer is honest, please choose the second option for this question”). The final sample consisted of 1,849 participants (59% female participants, mean age = 19.00, SD = 1.32, range = 17–23 years).

### 2.3. Measures

#### 2.3.1. Symptoms of problematic smartphone use

Problematic smartphone use symptoms were assessed using a modified version of the Internet Gaming Disorder diagnosis of nine items from the DSM-5 (28). An example item is “Do you always fail when attempting to cease or control your smartphone activity?”. The PSU scale has been used to measure PSU in several existing studies, with good internal consistency (Cronbach's alpha ranged from 0.85 to 0.86) (29–31). In addition, the scale sum score showed positive correlations with smartphone usage time across studies (29–31).

The scale measured nine symptoms, namely, immersion, withdrawal reactions, increased tolerance, self-control failure, loss of interest in other activities, recklessly continuing, driving to hide time spent, escaping a negative mood, and damaging society function. Participants were asked to report how often they had experienced each symptom over the past 12 months (ranging from 1 = “never” to 5 = “very often”). The Cronbach's alpha of this scale was .89 in the current study, indicating good internal consistency.

#### 2.3.2. Symptoms of depression

Depression symptoms were assessed by the Chinese version of the Patient Health Questionnaire-9 (PHQ-9) (32). This is a nine-item self-report measure based on the Diagnostic and Statistical Manual of Mental Disorders, Fourth Edition (DSM-IV), depression diagnostic criteria. The scale measured nine symptoms: anhedonia, depressed moods, sleep problems, fatigue, eating problems, hopelessness, concentration problems, psychomotor agitation/retardation, and suicide ideation. Participants were asked to report how often they had been bothered by each symptom over the past 2 weeks (ranging from 0 = “not at all” to 3 = “nearly every day”). The Cronbach's alpha of the PHQ-9 was .89 in the current study, indicating good internal consistency.

### 2.4. Data analysis

As an initial step, we examined the potential node redundancy (i.e., nodes that conceptually overlap with one another) before estimating the network. We followed the process described in recent research (33, 34). No potential node redundancy was screened out within the 18 examined variables (i.e., nine symptoms of PSU and nine symptoms of depression).

**Figure 1:**
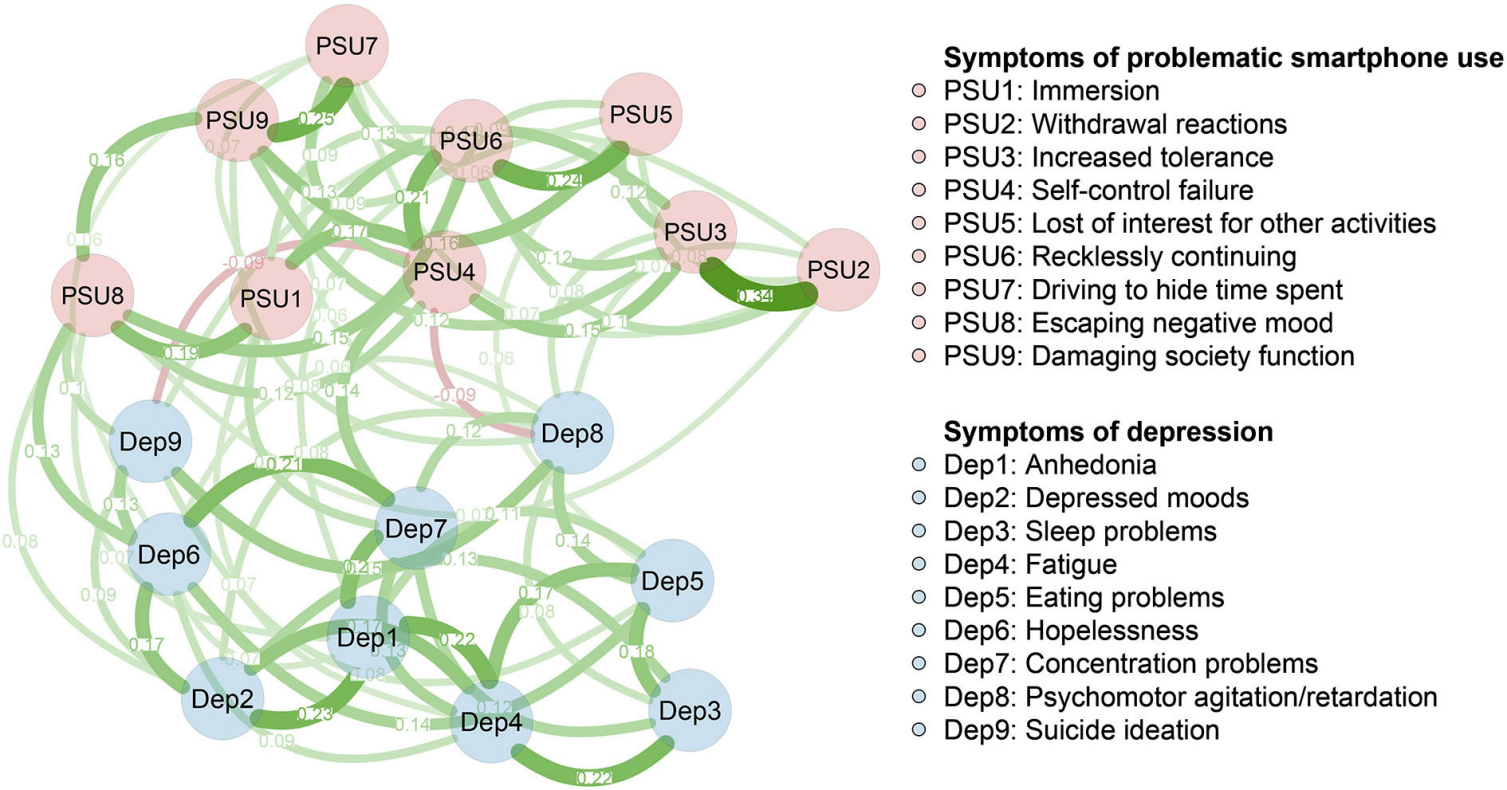
Network structure of different symptoms of problematic smartphone use and depression. Green edges represent positive correlations and red edges represent negative correlations. The thickness of the edge reflects the magnitude of the correlation. The value on the edge represented the unregularized partial correlation coefficient of two corresponding nodes.

We fitted a Gaussian graphical model (GGM) to the data (35). All symptoms of depression and PSU were depicted as nodes. An edge between any two symptoms represents a partial correlation between the two symptoms, after controlling for all other symptoms in the network. To account for the ordinal feature of the current data, we used Spearman correlations as input when constructing the network (36, 37). Due to the high sample size (n = 1849) and the expectation that many weaker bridging edges exist between symptoms of PSU and depression, we used unregularized model selection rather than regularization techniques commonly used in estimating GGMs (37, 38). Following recent recommendations (37), the current network estimation is based on the *ggmModSelect* method in the R-package *qgraph* (37, 39). The *Fruchterman–Reingold* algorithm was used for visualizing the layout (40). Within the presented network, positive correlations were depicted as green edges, while negative correlations were depicted as red edges. The magnitudes of correlations were reflected as edge thickness, with thicker edges representing stronger correlations. The value on the edge represented the unregularized partial correlation coefficient of two corresponding nodes in Figure 1. The steps were carried out using the R-package *qgraph* (39).

To identify the central nodes, we calculated the node expected influence (the sum of the edge weight of a given node) using the R-package *qgraph* (39). The higher the expected influence, the more central it is in the final network. Meanwhile, the other centrality indexes (i.e., strength, betweenness, and closeness) of each node were also calculated. To identify important nodes that bridge the PSU-Dep connection, we computed the bridge's expected influence (i.e., the sum of edge weights from a given node in one community to the other community) (16). Higher values of bridge expected influence represent a greater potential for increasing the risk of contagion to other communities (16). Two communities were pre-defined before analysis, namely the PSU symptom community (items from the PSU scale) and the depression symptom community (items from the PHQ-9 scale). The bridge expected influence was calculated using the R-package *networktools* (16).

Three steps were taken to ensure the accuracy and stability of the present network using the R-package *bootnet* (41). First, we bootstrapped (with 2,000 bootstrap samples) the 95% confidence interval of all edges within the network to ensure the accuracy of edge weights. Second, we computed the correlation stability (CS) coefficient of the bridge's expected influence to ensure the stability of this index. This is achieved through a case-dropping bootstrap approach (with 2,000 bootstrap samples). According to the recommendation, the ideal CS coefficient is above 0.5 and should not be below 0.25 (41). Third, we conducted bootstrapped difference tests (with 2,000 bootstrap samples) for edge weights and bridge expected influence.

We conducted a network comparison test (permutations = 1,000) to determine whether there are significant gender differences in terms of global strength (summed edge weights of the networks), edge weight, node expected influence, and bridge expected influence. The network comparison test was conducted using the R-package *NetworkComparisonTest* (42). Because we had no *a priori* hypotheses regarding differences in edges, corrections for multiple comparisons were not used when testing them in the present exploratory setting (42).

## 3. Results

### 3.1. Sample characteristics and descriptive statistics of variables

Table 1 shows the sample characteristics. Table 2 shows the mean scores and standard deviations for each variable selected in the present PSU-Dep network.

**Table 1:** Participants' characteristics (*N* = 1849).

**Variables**	**Mean (SD), Range, %**
Age	19.00 (1.32), 17–23
Female Gender	1094 (59.17%)
Only child	548 (29.64%)
Urban area	774 (41.86%)
**Educational level**	
First grade	1299 (70.25%)
Second grade	287 (15.52%)
Third grade	168 (9.09%)
Fourth grade	40 (2.16%)
Fifth grade	55 (2.97%)
Total score of depression	5.89 (4.09), 0–27
Total score of PSU	17.04 (6.06), 9–45

PSU, problematic smartphone use; SD, standard deviation.

**Table 2:** Abbreviation, mean scores and standard deviations for each variable selected in the present PSU-Dep network.

**Variables**	**Abbreviation**	**Mean (SD)**
**Symptoms of problematic smartphone use**		
Immersion	PSU1	2.65 (1.03)
Withdrawal reactions	PSU2	1.61 (0.82)
Increased tolerance	PSU3	1.78 (0.86)
Self-control failure	PSU4	2.15 (0.98)
Lost of interest for other activities	PSU5	1.61 (0.84)
Recklessly continuing	PSU6	1.84 (0.98)
Driving to hide time spent	PSU7	1.68 (0.85)
Escaping negative mood	PSU8	2.18 (1.10)
Damaging society function	PSU9	1.53 (0.78)
**Symptoms of depression**		
Anhedonia	Dep1	0.84 (0.59)
Depressed moods	Dep2	0.71 (0.57)
Sleep problems	Dep3	0.71 (0.73)
Fatigue	Dep4	0.85 (0.65)
Eating problems	Dep5	0.65 (0.66)
Hopelessness	Dep6	0.69 (0.66)
Concentration problems	Dep7	0.86 (0.70)
Psychomotor agitation/retardation	Dep8	0.39 (0.57)
Suicide ideation	Dep9	0.19 (0.45)

PSU, problematic smartphone use; Dep, depression; SD, standard deviation.

### 3.2. Symptom connections between PSU and depression

Figure 1 presents the 18-symptom PSU-Dep network. There are 17 of 81 (21%) between-community edges (weight range from−0.09 to 0.14) exhibited within the network. Overall, more positive (*n* = 15) than negative between-community edges (*n* = 2) were observed. These positive between-community edges are Dep1 (“anhedonia”)—PSU5 (“loss of interest in other activities”; edge weight = 0.07), Dep1 (“anhedonia”)—PSU8 (“escaping a negative mood”; edge weight = 0.07), Dep2 (“depressed mood”)—PSU8 (“escaping a negative mood”; edge weight = 0.08), Dep4 (“fatigue”) —PSU1 (“immersion”; edge weight = 0.07), Dep5 (“eating problems”)—PSU5 (“loss of interest for other activities”; edge weight = 0.06), Dep6 (“hopelessness”)—PSU7 (“driving to hide time spent”; edge weight = 0.06), Dep6 (“hopelessness”)—PSU8 (“escaping a negative mood”; edge weight = 0.13), Dep7 (“concentration problems”)—PSU4 (“self-control failure”; edge weight = 0.14), Dep7 (“concentration problems”)—PSU1 (“immersion”; edge weight = 0.10), Dep8 (“psychomotor agitation/retardation”)—PSU2 (“withdrawal reactions”; edge weight = 0.07), Dep8 (“psychomotor agitation/retardation”)—PSU3 (“increased tolerance”; edge weight = 0.08), Dep8 (“psychomotor agitation/retardation”)—PSU7 (“driving to hide time spent”; edge weight = 0.06), Dep8 (“psychomotor agitation/retardation”)—PSU9 (“damaging society function”; edge weight = 0.08), Dep9 (“suicidal ideation”) —PSU2 (“withdrawal reactions”; edge weight = 0.07), and Dep9 (“suicidal ideation”)—PSU8 (“escaping a negative mood”; edge weight = 0.10). The two negative between-community edges were Dep8 (“psychomotor agitation/retardation”)—PSU4 (“self-control failure”; edge weight = −0.09) and Dep9 (“suicidal ideation”)—PSU4 (“self-control failure”; edge weight = −0.09). The bootstrapped 95% confidence interval was relatively narrow, indicating that the PSU-Dep network is accurate (Supplementary Figure 1). Supplementary Figure 2 shows the bootstrapped difference test for edge weights.

### 3.3. Central symptoms

**Figure 2:**
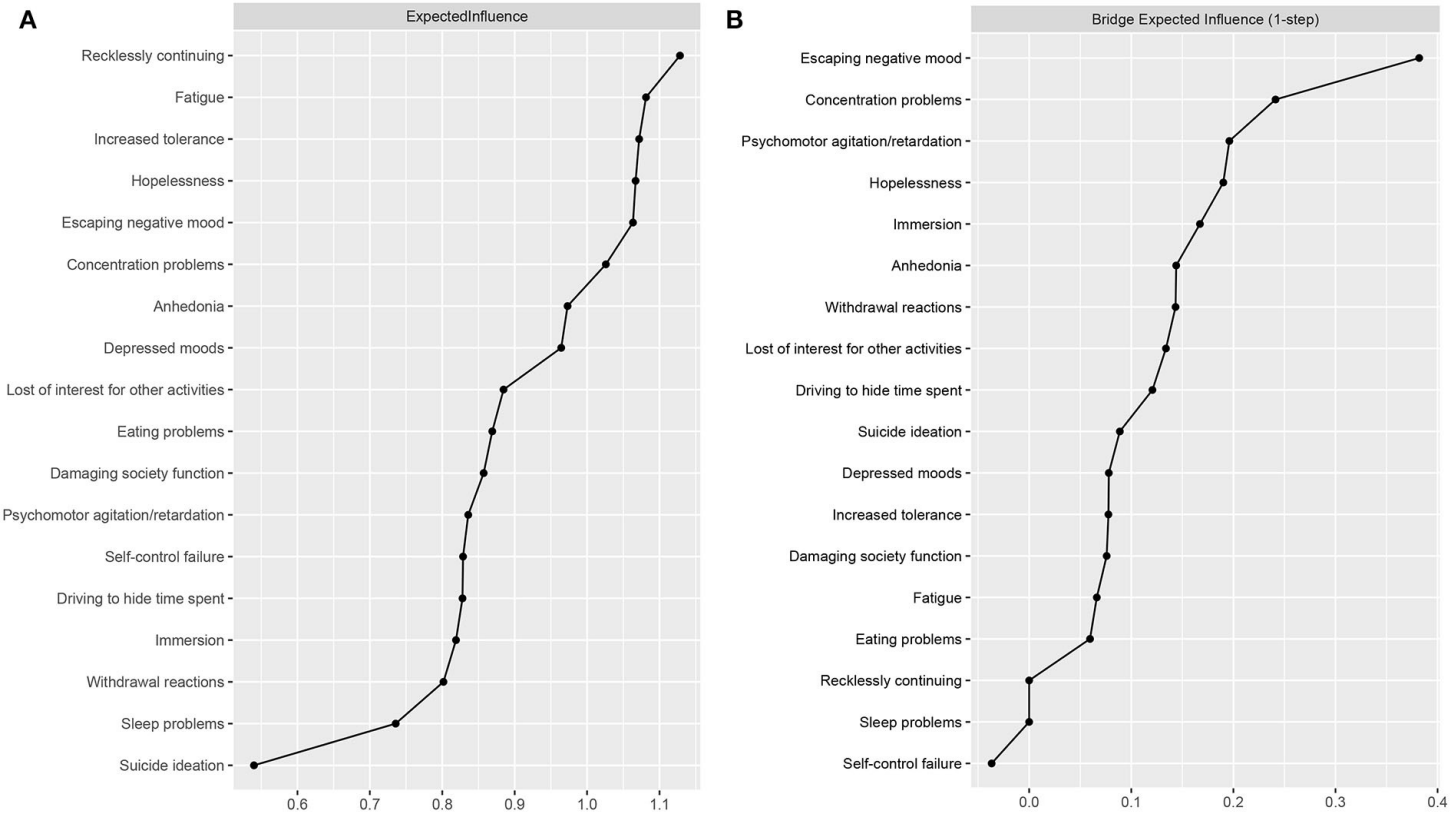
Centrality plot depicting the **(A)** expected influence and **(B)** bridge expected influence of each variable selected in the present network.

The expected influence values are shown in Figure 2A. PSU6 (“recklessly continuing”) and Dep4 (“fatigue”) showed high expected influence and were central nodes in the PSU-Dep network. The CS coefficient for node expected influence (value = 0.75) was larger than 0.5, indicating that this centrality index was adequately stable (Supplementary Figure 3). Supplementary Figure 4 shows the bootstrapped difference tests for node expected influence. Moreover, the results of other centrality indexes (i.e., strength, betweenness, and closeness) of each node can be found in Supplementary Figures 7–9.

### 3.4. Bridge symptoms

The bridge expected influence values are shown in Figure 2B. The two bridging nodes with the highest bridge expected influence were PSU8 (“escaping a negative mood”) and Dep7 (“concentration problems”). The CS coefficient for bridge expected influence (value = 0.59) was larger than 0.5, indicating that this centrality index was adequately stable (Supplementary Figure 5). Supplementary Figure 6 shows the bootstrapped difference tests for node bridge expected influence.

### 3.5. Gender differences in network characteristics

As for network global strength, we did not find significant gender differences (global strength [S] = 0.11, male participants = 8.37, female participants = 8.48, *p* = 0.63). There were significant differences between the two genders on two edges: PSU7 (“Driving to hide time spent”)—Dep6 (“hopelessness”; *p* = 0.03) and PSU8 (“escaping a negative mood”)—Dep1 (“anhedonia”; *p* = 0.01); both edges were stronger in male participants than in female participants. Moreover, there were no gender differences in the node expected influence and bridge expected influence of the PSU-Dep network.

## Discussion

Anchored from a network approach, this is the first study to uncover the network link between symptoms of PSU and depression in a large sample of young adults. The network model showed a small number of pathways between symptoms of PSU and depression and individual key symptoms in the symptom network. Globally, the network did not differ according to gender. The network model was statistically adequately accurate and stable. These findings may increase the present understanding of the association.

First, we found the link between some depression symptoms and PSU symptoms. Negative moods involving “anhedonia” and “depressed mood” are two important diagnostic symptoms for major depressive disorder (43) and are positively linked to “escaping a negative mood” for PSU; moreover, “anhedonia” for depression is positively linked to “loss of interest for other activities” for PSU. These findings in accordance with a compensatory internet use model show that negative emotions or moods lead to an escape motivation to indulge in smartphone use (44); and it adds to the model with a potential novel possibility that negative mood may not only motivate escape behaviors but also trigger “a loss of interest for other activities” symptom for PSU. Dysfunctional symptoms, such as “concentration problems” and “psychomotor agitation/retardation,” are important diagnostic symptoms for major depressive disorder (45); they are positively linked to some addictive symptoms of PSU involving mental distress or being functionally damaging. These findings are in accordance with the notion that mental dysfunction may be a cause of comorbidity of addiction and depression (46, 47). As previous studies have found, addiction and depression have similar manifestations of brain dysfunction (48). Our findings would inspire future research to explore whether depression and PSU have a common neuropsychological basis. “Hopelessness” and “suicidal ideation” for depression are important antecedents for suicidal behaviors (49). In our study, they were positively linked to “escaping a negative mood” for PSU; moreover, “hopelessness” for depression was positively linked to “driving to hide time spent” for PSU, and “suicide ideation” for depression was positively linked to “withdrawal reactions” for PSU. These findings in accordance with the compensatory internet use model show that a negative mood leads to an escape motivation to indulge in smartphone use (44). According to our findings, “driving to hide time spent” for PSU and “withdrawal reactions” for PSU may be valuable to be considered as predictors of suicidal-related psychology (hopelessness and suicidal ideation). In summary, these findings permeate deep into the essence of the link between PSU symptoms and depression symptoms for future researchers and practitioners to refer to.

Second, we found some central symptoms in the depression-PSU symptom network. “Recklessly continuing” for PSU and “fatigue” for depression were central symptoms in the joint network of PSU and depression. Furthermore, “recklessly continuing” was positively linked to all other PSU symptoms and not linked to any depression symptoms, indicating that it would be a key symptom within the PSU symptom network. This finding is in accordance with the notion that continued time consumption as an antecedent cause many other symptoms (e.g., immersion, increased tolerance, loss of interest in other activities) or a consequence caused by a few symptoms (e.g., self-control failure) (50, 51). Notably, according to this finding, further studies or clinical practice may be useful to consider the “recklessly continuing” symptom as a critical signal for the deterioration of other PSU symptoms. “Fatigue” for depression was positively linked with two-thirds of depression symptoms and one PSU symptom, showing that it was a central symptom within the depression symptom network. This result is in accordance with a recent network analysis study of depressive symptoms (52). Therefore, further studies or clinical practice may be valuable to consider “fatigue” as an important antecedent or consequence of other depression symptoms. In summary, these findings reveal the central symptoms in the joint symptom network of PSU and depression for future researchers and practitioners to refer to.

Finally, we found some bridge symptoms in the depression-PSU symptom network. “Escaping negative moods” for PSU and “concentration problems” for depression were bridge symptoms closely linked to the depression and PSU symptom networks, respectively. “Escaping negative mood” for PSU was significantly positively associated with many other depression symptoms (including “anhedonia,” “depressed mood,” “hopelessness,” and “suicide ideation”), implying that this symptom may be susceptible to or enhance negative affective symptoms of depression. The use of a smartphone to escape is likely to reduce the possibility of adopting positive emotional regulation (e.g., problem-solving and positive reappraisal) and increase negative emotional regulation (e.g., behavioral avoidance and expressive inhibition), which is associated with increased depression symptoms (53). However, these causal inferences would be carefully considered because some third-party factors (e.g., individuals' high-level motivation of escaping real life) may lead to a positive link between “escaping negative moods” for PSU and depressive symptoms. In sum, our findings emphasize that further studies or clinical practice may be useful to consider “escaping negative moods” for PSU as an important antecedent or consequence of depression symptoms or consider some third-party factors. The “concentration problems” for depression were significantly positively associated with “immersion” and “self-control failure” for PSU, indicating that people who have attention difficulty may be susceptible to immersing themselves in the smartphone world and have no control over their uses. In contrast, “immersion” and “self-control failure” toward smartphone use increase concentration-related depression symptoms. The former is in accordance with a recent longitudinal study that attention difficulty predicted the future severity of internet addiction (54). However, these causal inferences would be also carefully stated because some third-party factors (e.g., individuals' high-level motivation of escaping real life) may lead to a positive link between “concentration problems” for depression and “immersion” and “self-control failure” for PSU. Further studies or clinical practice may be valuable to consider “concentration problems” for depression as an important antecedent or consequence of PSU symptoms or consider some third-party factors. In summary, these findings reveal the bridge symptoms in the joint symptom network of PSU and depression for future researchers and practitioners to refer to.

This study has significant theoretical and practical implications. We focused on an emerging issue involving smartphone use and found two bridge symptoms (“escaping negative mood” for PSU and “concentration problems” for depression), which may provide a theoretical contribution. This contribution provides an opportunity to simplify the current theory involving this link. Specifically, a theory involving a pathway between PSU and depression may focus on a link between “escaping a negative mood” for PSU and affective symptoms of depression (e.g., depressed mood and anhedonia) or a link between “concentration problems” for depression and addictive symptoms for PSU (e.g., immersion and self-control failure). Furthermore, our findings contribute to theoretical and empirical studies involving this link that previously merely explored the relationship in terms of a total-score context. For example, depressed mood, an antecedent factor of PSU in theories (24), is only associated with a symptom related to escapism and not with any addictive symptoms in our study. Therefore, scholars or clinical practitioners may need to reconsider the link between depressive mood and behavioral addiction. In terms of practical implications, adjusting PSU to prevent depressive symptoms may be particularly vital in the age of smartphones. According to our findings, intervening in depression may be achieved efficiently by adjusting PSU. The first intervention approach is decreasing symptom connections between PSU and depression that might be appropriate for efficiently improving dangerous symptoms of depression (e.g., “suicidal ideation”) by adjusting and controlling for one or more related PSU symptoms (e.g., “withdrawal reactions”). The second is reducing the degree of bridge symptoms of PSU (i.e., “escaping a negative mood”), which logically may efficiently decrease the activated number of depression symptoms. For example, practitioners can focus on training coping strategies in individuals with depression to reduce the use of smartphones as a maladaptive coping strategy and promote more healthy coping strategies for managing negative moods. This may be especially suitable for individuals who have problems with both PSU and depression. Moreover, intervening in depression by reducing levels of a central symptom of depression (i.e., “fatigue”) connected to many other depression symptoms may be especially suitable for individuals who report many depressive symptoms.

Several limitations should be noted when interpreting current findings. First, the study used cross-sectional data. Therefore, a causal link between symptoms of PSU and depression cannot be determined. Further studies with a longitudinal design are required to illuminate the temporal sequence of symptoms between PSU and depression. Second, the PSU and depression scales that we used did not cover all symptoms, and it is difficult to include all symptoms in one study. Therefore, our findings may not be fully replicable by future studies using other scales involving other different symptoms. Third, the network structure in our non-clinical population may differ from that of clinically confirmed patients with depression. Future research can sample inpatients as participants. Fourth, the symptom “driving to hide time spent” may not be suitable for measuring the PSU among university students. Excessive smartphone use among university students is seldom supervised and controlled by others (55, 56). Therefore, future research may need to confirm the existence of this PSU symptom among adults.

## Conclusion

In conclusion, we aim to reveal a heterogeneous symptom-level connection between PSU and depression. Through symptom-level network analysis, we found (a) 17 important symptom connections between PSU and depression, (b) two central symptoms in depression and PSU, respectively, and (c) two bridge symptoms that are maximally linked with PSU symptoms and depression symptoms. These results provide a candidate answer as to why problematic information technology use is associated with depression from a symptom-level perspective. Future research can build on our findings to study the link between problematic information technology use symptoms and mental health disorder symptoms as a step toward a better public health environment.

## Data availability statement

The raw data supporting the conclusions of this article will be made available by the authors, without undue reservation. Requests to access these datasets should be directed to LR, rl_fmmu@163.com.

## Ethics statement

The studies involving human participants were reviewed and approved by the Ethics Committee of the First Affiliated Hospital of the Fourth Military Medical University (Project No. KY20202063-F-2). The questionnaire was completed after participants provided written informed consent.

## Author contributions

XW and LR developed the study ideas and designs. XW, FA, CL, KL, LW, LR, and XL wrote the original draft of this manuscript. All authors contributed to revising subsequent versions of the manuscript.
