# Supplementary material for: Escaping negative moods and concentration problems play bridge roles in the symptom network of problematic smartphone use and depression
Source: Front Public Health. 2023 Jan 17;10:981136. doi: 10.3389/fpubh.2022.981136 (PMC9886682; doi:10.3389/fpubh.2022.981136)
Supplement: Supplementary file 1 [file Data_Sheet_1.docx]

**Supplementary Materials**

1. Figure S1. Accuracy of edge weights
2. Figure S2. Bootstrapped difference test for edge weights
3. Figure S3. Stability of node expected influences
4. Figure S4. Bootstrapped difference test for node expected influences
5. Figure S5. Stability of node bridge expected influences
6. Figure S6. Bootstrapped difference test for node bridge expected influences
7. Figure S7. The results of other centrality indexes (i.e., strength, betweenness and closeness) of each node
8. Figure S8. Stability of node strengths (CS coefficient = 0.594).
9. Figure S9. Bootstrapped difference test for node strengths

Figure S1. Accuracy of edge weights

*Note*: The red line depicts the sample edge weights and the gray bar depicts the bootstrapped confidence interval.

Figure S2. Bootstrapped difference test for edge weights

*Note*: Gray boxes indicate edge weights that do not differ significantly from one another, while black boxes indicate edge weights that do differ significantly. Blue and red boxes on the diagonal correspond to edge weights with positive and negative correlations, respectively. The text of PSU and Dep can be seen in Table 2.

Figure S3. Stability of node expected influences

*Note*: The red bar represents the average correlation between node expected influences in the full sample and subsample with the red area depicting the 2.5th quantile to the 97.5th quantile.

Figure S4. Bootstrapped difference test for node expected influences

*Note*: Gray boxes indicate node expected influences that do not differ significantly from one another, while black boxes indicate node expected influences that do differ significantly. The number in the white boxes (i.e., diagonal line) represent the value of node expected influences. The text of PSU and Dep can be seen in Table 2.

Figure S5. Stability of node bridge expected influences

*Note*: The red bar represents the average correlation between node bridge expected influences in the full sample and subsample with the red area depicting the 2.5th quantile to the 97.5th quantile.

Figure S6. Bootstrapped difference test for node bridge expected influences

*Note*: Gray boxes indicate node bridge expected influences that do not differ significantly from one another, while black boxes indicate node bridge expected influences that do differ significantly. The text of PSU and Dep can be seen in Table 2.

Figure S7. Centrality plot depicting the strength, betweenness and closeness of each node.

Figure S8. Stability of node strengths (CS coefficient = 0.594).

*Note:* The red bar represents the average correlation between node strengths in the full sample and subsample with the red area depicting the 2.5th quantile to the 97.5th quantile.

Figure S9. Bootstrapped difference test for node strengths

*Note*: Gray boxes indicate node strengths that do not differ significantly from one another, while black boxes indicate node strengths that do differ significantly. The text of PSU and Dep can be seen in Table 2.
